# High‐Dose Tramadol Enhances the Proliferative and Invasive Potential of Pancreatic Ductal Adenocarcinoma in Mice Through Microenvironmental Alteration

**DOI:** 10.1155/prm/6594413

**Published:** 2026-06-22

**Authors:** Tomoya Kuramochi, Tomoaki Itaya, Makoto Sano, Yukino Oshima, Jinsuk Kim, Osamu Kitajima, Hideaki Ijichi, Takahiro Suzuki

**Affiliations:** ^1^ Department of Obstetrics and Gynecology, Juntendo University, Urayasu Hospital 2-1-1 Tomioka, Urayasu, Chiba, 279-0021, Japan, juntendo.ac.jp; ^2^ Department of Anesthesiology, Nihon University School of Medicine, 30-1 Oyaguchi-Kamimachi Itabashi-ku, Tokyo, 173-8610, Japan, nihon-u.ac.jp; ^3^ Department of Gastroenterology, The University of Tokyo, 7-3-1 Hongo Bunkyo ku, Tokyo, 113-0033, Japan, u-tokyo.ac.jp; ^4^ Clinical Nutrition Center, Graduate School of Medicine, The University of Tokyo, 7-3-1 Hongo Bunkyo ku, Tokyo, 113-0033, Japan, u-tokyo.ac.jp

**Keywords:** CD8^+^T lymphocytes, *KPPC* mouse, M2-like tumor-associated macrophages, pancreatic cancer, tramadol, weak opioid

## Abstract

**Purpose:**

Opioids are known to have various effects on cancer depending on the type and dose. Tramadol, a weak opioid used for mild cancer pain, exhibits antitumor effects, whereas strong opioids demonstrate protumor effects. We previously reported that 10 mg/kg/day of tramadol (low dose) improves cancer pain and exerts antitumor effects in mice with pancreatic cancer. However, the effects of high‐dose tramadol on pancreatic cancer remain unknown. Therefore, the effect of high‐dose tramadol on pancreatic cancer was investigated in mice using the pancreatic cancer model mouse *KPPC* (*L* *S* *L* − *K* *r* *a* *s* ^*G*12*D*/+^;  *T* *r* *p*53^*f* *l* *o* *x*/*f* *l* *o* *x*^;  *P* *d* *x* − 1^*c* *r* *e*/+^).

**Methods:**

High‐dose tramadol (50 mg/kg/day) was orally administered to 6‐week‐old *KPPC* mice bearing pancreatic ductal adenocarcinoma until a humane endpoint (*n* = 10). Cancer‐related pain was assessed using the mouse grimace scale, and tumor status was determined histopathologically. Plasma cytokine concentrations were assessed using a cytokine array. The effects of tramadol on the invasive potential of murine pancreatic ductal adenocarcinoma cell lines were investigated in vitro.

**Results:**

High‐dose tramadol improved mouse grimace scale scores but increased tumor size (1734.2 vs. 907.1 mm^2^; *P*  <  0.01). High‐dose tramadol stimulated proliferative Ki‐67 labeling index and inhibited local infiltration of CD8^+^ cytotoxic T cells and M2‐like tumor‐associated macrophages. Similar to alterations in local immunoinflammatory cells and relief of cancer pain, plasma tumor necrosis factor‐*α*, interleukin (IL)‐6, IL‐2, IL‐12, and interferon‐*γ* decreased following high‐dose tramadol administration. The invasive potential of mouse pancreatic ductal adenocarcinoma cell lines was suppressed by adding high‐dose tramadol.

**Conclusion:**

These results suggest that high‐dose tramadol improves cancer‐associated pain but enhances the tumor volume of pancreatic ductal adenocarcinoma by decreasing anti‐tumor CD8^+^ T lymphocytes.

## 1. Introduction

Pancreatic ductal adenocarcinoma (PDAC) is a prominent and aggressive subtype of pancreatic cancer [1]. Around two‐thirds of patients experience pain in the early stages of PDAC, whereas more than 80% of patients with PDAC experience pain in advanced stages [2]. As such, appropriate anesthetics are required for the management of patients with PDAC‐associated pain.

Tramadol (TRA), a weak opioid, is used as the second step in the World Health Organization (WHO) analgesic ladder for patients with cancer, including PDAC [3]. The analgesic potency of TRA is approximately one‐tenth that of the strong opioid morphine when administered orally [4]. However, TRA has been shown to exert antitumor effects against several cancers, such as lung [5], breast [6], and pancreatic cancers [7]. By comparison, strong opioids, such as morphine, enhance or inhibit tumor growth [8]. Immunosuppression and other cancer regulatory effects, such as increased tumor angiogenesis and activation of antiapoptotic pathways, are primarily considered to be associated with tumor promotion. In contrast, immunoactivation via blocking cancer pain by analgesics is mainly associated with tumor suppression. However, the detailed mechanisms by which opioids exert these effects are likely complex and differ according to type and concentration [8, 9].

We previously reported that 10 mg/kg/day of TRA (low dose) improves cancer‐associated pain in mice with PDAC and inhibits PDAC by increasing antitumor M1‐like tumor‐associated macrophages (TAMs) [7]. In patients with non‐cancer‐related disease, TRA exhibits anti‐inflammatory properties and can retain immune cells such as natural killer (NK) cells [10]. Conversely, strong opioids, including morphine and fentanyl, inhibit CD8^+^ T cells [9] and NK cells [11], thereby promoting tumorigenesis. The effects of TRA on cancer growth are weaker than those of strong opioids. This is because TRA’s effect on µ‐opioid receptors is not as strong as that of strong opioids, and TRA is involved in the descending pain inhibitory system via serotonin and noradrenaline receptors. Administration of 50 mg/kg/day of TRA (defined as high‐dose TRA) induces *µ*‐opioid stimulation similar to that of strong opioids. We hypothesized that administration of high‐dose TRA has cancer‐promoting effects similar to those observed with administration of strong opioids. We further hypothesized that the mechanism involves changes in the immune‐inflammatory microenvironment, including the localization of TAMs, in pancreatic cancer.

In our previous paper, low‐dose TRA showed antitumor effects on pancreatic cancer with increasing local antitumor M1‐like TAMs [7]. In the present paper, meanwhile, high‐dose TRA was found to promote pancreatic cancer with alteration of tumor microenvironments, such as a decrease of local CD8^+^ lymphocytes. For this study, *KPPC* (*L* *S* *L* − *K* *r* *a* *s* ^*G*12*D*/+^;  *T* *r* *p*53^*f* *l* *o* *x*/*f* *l* *o* *x*^;  *P* *d* *x* − 1^*c* *r* *e*/+^) mice, a spontaneous PDAC mouse model with an innate immune‐inflammatory system, were used [12, 13].

## 2. Materials and Methods

### 2.1. Study Design and Ethical Approval Statement

This was a preclinical, in vivo, experimental study (interventional, nonclinical) and an in vitro, experimental study (nonclinical). The study protocol followed the Animal Research: Reporting of In Vivo Experiments (ARRIVE) guidelines. The study was approved by the Nihon University School of Medicine Animal Care and Use Committee (AP21MED013 and AP22MED069).

### 2.2. Reagents

TRA was obtained from Sigma‐Aldrich Japan (Tokyo, Japan) and diluted in distilled water. The sterile water was used for the animal experiments.

### 2.3. TRA Administration to *KPPC* Mice

Six‐week‐old spontaneous PDAC model mice, *KPPC* (*L* *S* *L* − *K* *r* *a* *s* ^*G*12*D*/+^;  *T* *r* *p*53^*f* *l* *o* *x*/*f* *l* *o* *x*^;  *P* *d* *x* − 1^*c* *r* *e*/+^), were randomly divided into two groups. One was orally administered 50 mg/kg/day of TRA (TRA50; *n* = 10) until the humane endpoint was reached (no activity, including grooming, food intake of < 1 g/day, or > 20% body weight loss within several days). The other was administered vehicle instead of TRA (vehicle; *n* = 12). 6‐week‐old healthy control (Cre‐negative) littermate mice were administered 50 mg/kg/day of TRA (TRA50; high dose; *n* = 4) until 70 days of age. Mice received TRA as a spontaneous intake. Body weight and water intake were measured daily, and the TRA intake was daily adjusted to 50 mg/kg/day in TRA50 group. All animals were housed under pathogen‐free conditions with free access to food and water before euthanasia. Only the experiment supervisor knew which group each mouse belonged to during TRA administration to the mice and in the subsequent analysis. No cases were excluded in each experimental group. The vehicle mouse population consists of eight males and four females. The TRA50 mouse population consists of four males and six females. Also, because the mice were used in the experiment in the order they were born, there are individual differences in sex.

### 2.4. Pain Analysis

The mouse grimace scale (MGS) (score of 0–2 each; total score of 10) was used to evaluate cancer‐related pain, as previously reported [7, 12, 13]. The MGS assesses pain based on the mouse’s facial expression and ear and whisker tilt. Therefore, it is suitable for evaluating spontaneous cancer pain and the effects of analgesics such as TRA on it. The MGS was measured simultaneously by two blinded investigators, and the average value was recorded.

### 2.5. Mouse Autopsy

At the humane endpoint, all mice were euthanized by carbon dioxide (using a 10‐L container, administered at 7 L/min for 5 min) and autopsied. Blood samples were collected from the right atrium using a 27G needle at the humane endpoint. The total pancreatic tumor volumes were calculated using the formula *π*/6 × width × length × height. The weight of the pancreas was measured for the entire pancreas, including the adjacent duodenum (approximately 1.5 cm in size), to assess tumor infiltration into the duodenum.

### 2.6. Immunohistochemistry

Immunostaining of pancreatic tumors was performed using antibodies (Supporting Table 1), except for cases in which mice were found dead or in cases of tumors with massive ischemic central necrosis (Supporting Table 2). Cases with unclear staining of each internal control were excluded. The number of positive cells in a typical specimen was determined in at least three fields (200×). The number of positive cells in each category was measured using ImageJ software (National Institutes of Health, USA).

### 2.7. Cytokine Antibody Array

Except for icteric and hemolytic samples, pooled plasma samples from *KPPC* mice treated with TRA50 (*n* = 6), vehicle‐treated *KPPC* mice (*n* = 4), and healthy mice (*n* = 4) were examined twice using the RayBio^®^ C‐Series Mouse Cytokine Antibody Array 1000 (RayBiotech, Inc., Norcross, GA, USA). Cases with dead found, hemolysis, or severe jaundice were excluded from the measurement (Supporting Table 2). A total of four spots were measured according to the manufacturer’s instructions.

### 2.8. Invasion Assay

Murine PDAC cell lines (#146, 244) were previously established from *K* *P* *C* ^*f* *l* *o* *x*^ (*L* *S* *L* − *K* *r* *a* *s* ^*G*12*D*/+^;  *T* *r* *p*53^*f* *l* *o* *x*/+^;  *P* *d* *x* − 1^*c* *r* *e*/+^) mice [14]. Cells (2.5 × 10^4^ cells) were plated in Matrigel‐coated invasion chamber inserts in 0.5 mL of serum‐free Dulbecco’s modified Eagle medium (DMEM) (Corning Life Sciences, Tokyo, Japan). Phosphate‐buffered saline (PBS (−); Nissui Pharmaceutical Co., Tokyo, Japan) was used as the vehicle. The inserts were then placed in wells with 0.75 mL of DMEM containing 10% fetal bovine serum with/without 30 µM TRA or 100 µM TRA each and incubated for 24 h. The TRA concentration was determined based on the concentrations in the previous paper [15]. The number of cells in a typical specimen was determined in at least four fields (100×). The number of positive cells in each category was measured using ImageJ software.

### 2.9. Statistical Analysis

The sample size was determined based on our previous studies conducted in the past [7, 12]. For comparison of Kaplan–Meier survival curves, univariate survival analyses with the log‐rank test were used. Analyses of tumor volume, histology, and invasiveness were performed using EZR (Easy R) software, version 1.55, using the parametric Student′s *t*‐test, Tukey–Kramer test, nonparametric Mann–Whitney *U* test, or Steel–Dwass test, as appropriate [7, 13, 16]. A *p* value of < 0.05 indicated significance.

## 3. Results

### 3.1. High‐Dose TRA Improved Cancer‐Associated Pain by Decreasing Inflammatory Cytokines

**FIGURE 1:**
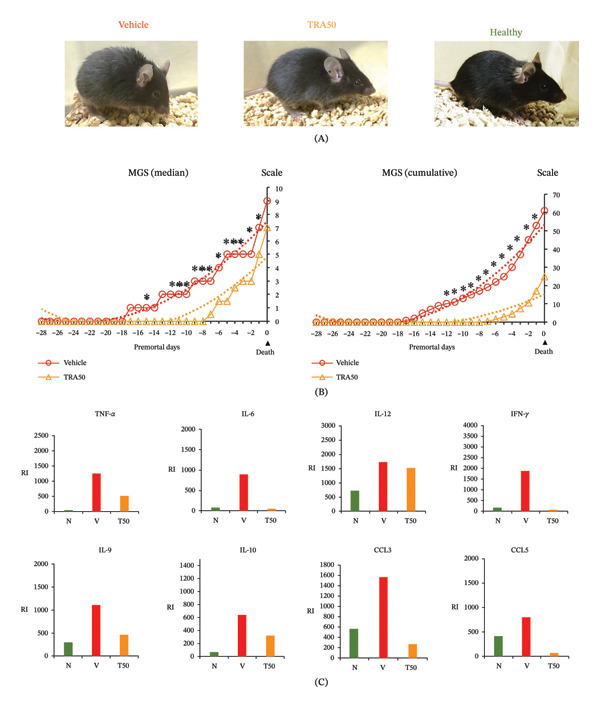
Inhibition of cancer‐associated pain and plasma cytokine levels in TRA‐treated *L* *S* *L* − *K* *r* *a* *s* ^*G*12*D*/+^; *T* *r* *p*53^*f* *l* *o* *x*/*f* *l* *o* *x*^; *P* *d* *x* − 1^*c* *r* *e*/^ *+* (*KPPC*) mice. (A) Representative image of a vehicle‐treated *KPPC* mouse with pain signs including a severe nose and cheek bulge (MGS 8). *KPPC* mouse treated with TRA at 50 mg/kg/day (TRA50) (MGS 4). No sign of pain in the healthy mouse. (B) Median and cumulative MGS scores for *KPPC* mice treated with TRA50 (*n* = 10), and vehicle water (*n* = 12). *p*  <  0.05 by the nonparametric Mann–Whitney *U* test (∗, TRA50 vs. vehicle). (C) Pooled plasma levels of cytokines in normal control (N; *n* = 4) and *KPPC* mice treated with TRA50 (T50; *n* = 6) or vehicle water (V; *n* = 4). Data are presented as the mean of four array spots. CCL, CC chemokine ligand; IFN, interferon; IL, interleukin; MGS, mouse grimace scale; RI, relative intensity; TNF, tumor necrosis factor; TRA, tramadol.

We previously reported that low‐dose TRA (TRA10) improved cancer‐associated pain in mice with PDAC. Here, we investigated the analgesic effects of high‐dose TRA (TRA50) using the same PDAC mouse model. As expected, TRA50 considerably reduced pain signs, including nose and cheek bulging (Figure 1A). Moreover, TRA50 significantly improved the median MGS scores at 1–12 premortal days (pmds) and 15 pmds (*p* = 0.02) compared with vehicle (Figure 1B). Similarly, cumulative MGS scores during the 1–12 pmds period were significantly lower in TRA50 mice than in vehicle‐treated mice (Figure 1B). In healthy mice, the MGS score remained low throughout the observation period (Supporting Figure 1a and 1b).

Next, we investigated plasma cytokine levels, given that a decrease in inflammatory cytokines can improve inflammatory nociceptive pain (Figure 1C). Interestingly, levels of the major nociceptive pain‐related cytokines, including tumor necrosis factor (TNF)‐*α* and interleukin (IL)‐6, were lower in the TRA50 group than in the vehicle‐treated group (Figure 1C). Plasma levels of the cytokines IL‐12 and IFN‐*γ*, which are secreted by CD8 cells and promote antitumor effects [17], increased in the vehicle‐treated group but decreased in the TRA50 group. In addition, IL‐9 and IL‐10, which are released by IL‐9‐producing CD8^+^ T (Tc9) cells that differentiate from CD8 cells and exert antitumor effects [18], increased in the vehicle group but decreased in the TRA50 group. Indeed, levels of CC chemokine ligand (CCL)3 and CCL5, which are related to cancer‐associated fibroblasts (CAFs), were lower in TRA50‐treated mice than in vehicle‐treated mice (Figure 1C).

### 3.2. High‐Dose TRA Increased Tumor Volume

**FIGURE 2:**
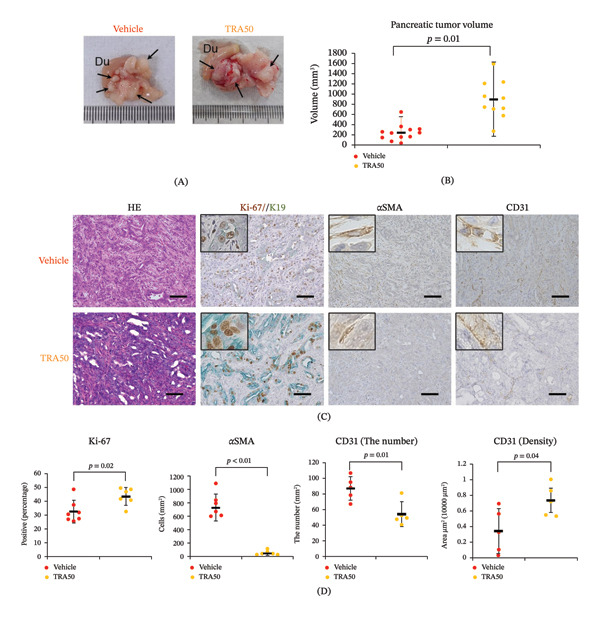
Effects of TRA on PDAC in *KPPC* mice. (A) Gross appearance (arrows, tumor nodules; Du, duodenum). (B) Volume of the pancreatic tumor in *KPPC* mice treated with TRA at 50 mg/kg/day (TRA50) (*n* = 10) or vehicle water (*n* = 12). Data are presented as mean values. TRA50 (*n* = 10) versus vehicle (*n* = 12) (95% CI 109.8 to 718.5; *p* = 0.01) by the parametric Student′s *t*‐test. (C) Histopathological features including HE staining. Ki‐67 in K‐19^+^ PDAC is greater in TRA50‐treated mice than in vehicle‐treated mice, whereas stromal *α*‐SMA^+^ juxta‐tumoral CAFs are decreased in the TRA50‐treated group. Micro vessel density of CD31^+^ increases, whereas the number of CD31^+^ decreases in the TRA50‐treated group. Insets, 200× original magnification. Scale bars, 100 µm. The enlarged view in the upper left is 400× original magnification. (D) Quantification of the staining in (C). Data are presented as mean ± SD (normality data) or median ± IQR (non‐normality data). Ki‐67: TRA50 (*n* = 6) versus vehicle (*n* = 7) (95% CI 1.8 to 20.0; *p* = 0.02) by the parametric Student′s *t*‐test. *α*‐SMA: TRA50 (*n* = 6) versus vehicle (*n* = 6) (*p*  <  0.01) by the nonparametric Mann–Whitney *U* test. Number of CD31: TRA50 (*n* = 5) versus vehicle (*n* = 5) (95% CI −55.0 to −9.9; *p* = 0.01) by the parametric Student′s *t*‐test. Density of CD31: TRA50 (*n* = 5) versus vehicle (*n* = 5) (95% CI 0.03 to 0.76; *p* = 0.04) by the parametric Student′s *t*‐test. *α*‐SMA, *α*‐smooth muscle actin; HE, hematoxylin‐eosin; K19, cytokeratin 19; PDAC, pancreatic ductal adenocarcinoma; TRA, tramadol.

To investigate the effects of TRA50 on PDAC, autopsies were performed at the humane endpoint. Solid and whitish tumor nodules were observed in the pancreas of TRA50‐treated *KPPC* mice (Figure 2A and Supporting Table 2). Tumor volume was greater in TRA50‐treated mice than in vehicle‐treated mice (1734.2 vs. 907.1 mm^2^; *p* < 0.01) (Figure 2B). No difference was observed in the weight of the pancreas and the adjacent duodenum (1287.6 vs. 1395.8 mg; *p* = 0.66) (Supporting Figure 1C). Food intake was higher in TRA50‐treated mice than in vehicle‐treated mice (Supporting Figure 2a and 2b), but there was no significant difference in body weight (Supporting Figure 2). There was no significant difference in median survival time (TRA50 vs. vehicle, 61.5 vs. 64.0 days; *p* = 0.78) on Kaplan–Meier analysis (Supporting Figure 3). Histologically, the proliferative Ki‐67 labeling index of epithelial K‐19^+^ PDAC in *KPPC* mice was higher in those treated with TRA50 than in those treated with vehicle (43.5% vs. 32.6%; *p* = 0.02) (Figure 2C and D). CAFs, which are positive for *α*‐smooth muscle actin (*α*‐SMA), are involved in malignant transformation and treatment resistance of pancreatic cancer [19]. Local *α*‐SMA^+^ CAFs in TRA50‐treated mice decreased compared with vehicle‐treated mice (52.4 vs. 730.8 cells/mm^2^; *p*  <  0.01) (Figure 2C and D). The number of CD31^+^ tumor blood vessels decreased in TRA50 mice (TRA50 vs. vehicle, 55.0 vs. 87.5/mm^2^; *p* = 0.01), but the microvessel density increased considerably in mice treated with TRA50 (TRA50 vs. vehicle, 0.74 vs. 0.34/mm^2^; *p* = 0.04) (Figure 2C and D).

### 3.3. High‐Dose TRA Inhibited Local CD8^+^ T Cell Infiltration

**FIGURE 3:**
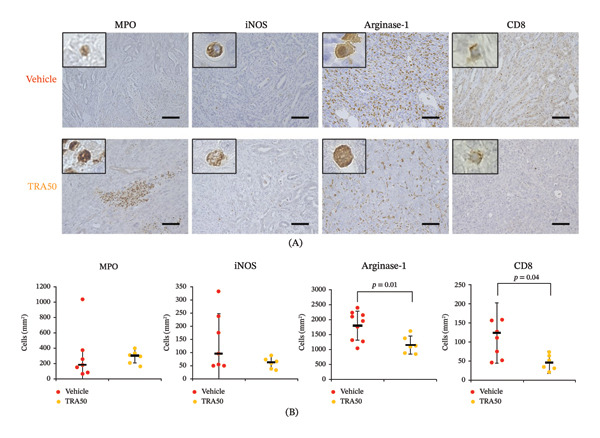
Effects of TRA on inflammatory cell infiltration in PDAC mice. (A) There are no differences between the TRA50‐treated group and vehicle‐treated group in MPO^+^ cells and iNOS^+^ M1‐like TAMs. Arginase1^+^ M2‐like TAMs are decreased in the TRA group. CD8^+^ cells are lower after TRA50 than in the vehicle‐treated group. Insets, 200× original magnification. Scale bars, 100 µm. The enlarged view in the upper left is 400× original magnification. (B) Quantification of the staining in (A). Data are presented as mean ± SD (normality data) or median ± IQR (non‐normality data). MPO: TRA50 (*n* = 6) versus vehicle (*n* = 7) (*p* = 0.37) by the nonparametric Mann–Whitney *U* test. iNOS: TRA50 (*n* = 6) versus vehicle (*n* = 7) (*p* = 0.22) by the nonparametric Mann–Whitney *U* test. Arginase‐1: TRA50 (*n* = 6) versus vehicle (*n* = 9) (95% CI −1116.6 to −161.3; *p*  <  0.01) by the parametric Student′s *t*‐test. CD8: TRA50 (*n* = 6) versus vehicle (*n* = 7) (*p*  <  0.04) by the nonparametric Mann–Whitney *U* test. iNOS, inducible nitric oxide synthase; MPO, myeloperoxidase; PDAC, pancreatic ductal adenocarcinoma; TAM, tumor‐associated macrophage; TRA, tramadol.

To understand the pathogenesis of tumor proliferation and invasion, as well as changes in cytokine levels, characteristics of the local stromal microenvironment, such as immunoinflammatory cell infiltration, were investigated. Notably, the high‐dose TRA group showed fewer CD8^+^ cells than the vehicle group (43.6 vs. 111.1 cells/mm^2^; *p* = 0.04) (Figure 3A and B). However, TRA50 administration decreased arginase‐1^+^ tumor‐promoting M2‐like TAMs (TRA50 vs. vehicle, 1150.1 vs. 1795.3 cells/mm^2^; *p* = 0.01), but no significant difference was seen in inducible nitric oxide synthase+ (iNOS^+^) antitumor M1‐like TAMs and proinflammatory myeloperoxidase+ (MPO^+^) tumor‐associated neutrophils (TANs) (Figure 3A and B).

### 3.4. High‐Dose TRA Inhibited Invasion of PDAC Cells In Vitro

**FIGURE 4:**
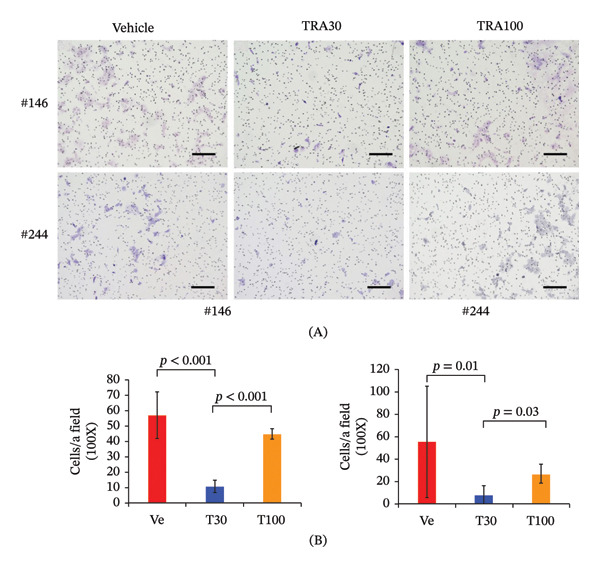
Invasion assay of PDAC in vitro. (A) PDAC cells are inoculated with 30 µM of TRA (T30), 100 µM of TRA (T100), or vehicle (Ve). Invasive cells are detected using Giemsa staining. 100× original magnification. Scale bars, 100 µm. (B) Quantification of the staining in (A). Data are presented as mean ± SD (normality data) or median ± IQR (non‐normality data). #146: T100 (*n* = 4) versus T30 (*n* = 4) (95% CI −50.4 to −17.6; *p*  <  0.001); T100 (*n* = 4) versus Ve (*n* = 4) (95% CI −2.6 to 30.1; *p* = 0.10); T30 (*n* = 4) versus Ve (*n* = 4) (95% CI 31.4 to 64.1; *p*  <  0.001) by the parametric Tukey–Kramer test. #244: T100 (*n* = 6) versus T30 (*n* = 6) (*p* = 0.03); T100 (*n* = 6) versus Ve (*n* = 12) (*p* = 0.15); T30 (*n* = 6) versus Ve (*n* = 1P2) (*p* = 0.01) by the nonparametric Steel–Dwass test. PDAC, pancreatic ductal adenocarcinoma; TRA, tramadol.

There was no significant increase in PDAC invasion or metastasis in the TRA50‐treated group compared with the vehicle‐treated group (Supporting tables 3 and 4). In vitro experiments showed that low concentrations of TRA suppressed tumor invasion of two different PDAC cell lines (#146 and #244), whereas high doses of TRA enhanced the invasion potential (#146; vehicle vs. TRA30 vs. TRA100, 60.0 vs. 10.8 vs. 44.8 cells/HPF; vehicle vs. TRA30; *p*  <  0.001, TRA30 vs. TRA100; *p*  <  0.001, #244: vehicle vs. TRA30 vs. TRA100, 62.5 vs. 6.0 vs. 26.0 cells/HPF; vehicle vs. TRA30; *p* = 0.01, TRA30 vs. TRA100; *p* = 0.03) (Figure 4A and B).

## 4. Discussion

**FIGURE 5:**
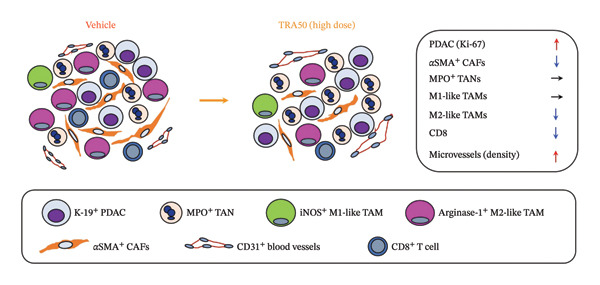
Hypothetical mechanism of high‐dose TRA’s effects on immune regulation in PDAC. Mice treated with TRA at 50 mg/kg/day (TRA50) show a higher Ki‐67 index of PDAC and lower local CD8^+^ cells and M2‐like TAMs compared with vehicle‐treated mice. *α*‐SMA, *α*‐smooth muscle actin; CAF, cancer associated fibroblast; TAN, tumor‐associated neutrophil; PDAC, pancreatic ductal adenocarcinoma; TAM, tumor‐associated macrophage; TRA, tramadol.

The current study showed that high‐dose TRA significantly improved cancer‐associated pain but promoted an increase in tumor volume of PDAC in vivo. Furthermore, in the tumor microenvironment, an increase in Ki‐67 and a decrease in CD8 were observed (Figure 5). Ki‐67 is a nuclear protein that plays a crucial role in cell proliferation and ribosomal RNA transcription. Its expression is observed during the G1, S, G2, and M phases of the cell cycle, but not during the quiescent G0 phase [20]. Therefore, Ki‐67‐positive cells indicate active cell proliferation. Furthermore, Ki‐67 has been shown to correlate with recurrence risk and prognosis in PDAC [20]. CD8 is expressed on the surface of cytotoxic T cells and suppressor T cells that act suppressively against cancer [18]. These changes in Ki‐67 and CD8 in the TRA50 group suggest that high‐dose TRA promotes the proliferation of PDAC. On the other hand, aSMA, which is involved in treatment resistance, decreased [21]. In vitro studies showed an increase in invasion of PDAC cells. These results showed a mix of effects, with some aspects suppressing pancreatic cancer and others promoting it. This may be related to the fact that TRA affects PDAC through multiple pathways. There are multiple pathways by which opioids, including TRA, can affect cancer [22]. The first pathway involves activation of the immune system, which has an antitumor effect, by suppressing cancer pain [23]. The second pathway involves opioid activation of the immune responses associated with tumor growth [23]. The third pathway involves direct effects of the opioids themselves on tumors [23]. Improvement of cancer‐associated pain generally stimulates the immune system and suppresses the release of endogenous opioids, thereby blocking tumor growth [8, 23]. In this case, the reduction in cancer pain may be involved in reducing tumor size. Our previous report showed that TRA also inhibits the proliferation of PDAC cell lines in a dose‐dependent manner in vitro [7]. Therefore, the antitumor effect of TRA itself reduces tumor size. However, the mechanism that increases tumor growth via microenvironmental factors is promoted. Overall, the balance among these three pathways may result in no tumor growth with administration of low‐dose TRA but an increase in tumor growth with administration of high‐dose TRA.

The effects of strong opioids, such as morphine, inhibit the function of CD8^+^ T cells [9] and NK cells [11], thereby promoting tumor growth. Our model mice had few NK cells, even in the vehicle‐treated group; however, mice treated with high‐dose TRA showed a decrease in local antitumor CD8^+^ T cells. Numbers of M2‐like TAMs decreased in both the low‐dose TRA group, which we reported previously [7], and the high‐dose TRA group, whereas low‐dose TRA stimulated local recruitment of anti‐tumor M1‐like TAMs [7]. However, the current study also found that high‐dose TRA did not stimulate local recruitment of M1‐like TAMs. This result suggests that TRA may change the type of TAMs depending on the dose. In addition, the number of MPO^+^ TANs in tumors was increased by TRA50 administration in this study compared with low‐dose TRA reported in previous studies [7]. MPO is found in the cytoplasm of neutrophils. MPO^+^ TANs are known to be involved in cancer proliferation [24]. TRA may also affect the number of MPO^+^ TANs depending on the dose. The reduction in CAFs in the high‐dose TRA group was also observed in the low‐dose TRA group [7], which may have been related to pain reduction [19]. The decreases in TNF‐*α*, IL‐6, CCL3, and CCL5 induced by high‐dose TRA might also be attributable to the decrease in CAFs [25–27]. Furthermore, decreases in TNF‐*α*, IL‐6, IL‐10, and CCL3 suggest a decrease in M2‐like TAMs (Supporting Figure 3A) [28]. Decreases in IL‐12, IFN‐*γ*, IL‐9, and IL‐10 may be associated with a decrease in CD8^+^ T cells and Tc9 cells that differentiate from them [17], which are considered to have antitumor effects [18]. However, previous studies using low‐dose TRA have shown a reduction in tumor invasion [7], whereas the current study did not show a reduction in tumor invasion compared with the vehicle‐treated group. The present study found that high‐dose TRA enhanced the invasive potential of PDAC cell lines in vitro. The reversal of the reduced invasion potential of PDAC might have been a direct effect of high‐dose TRA.

This research has several limitations. The first limitation concerns the weight of the pancreas. Although the tumor volume increased with high‐dose TRA administration, no difference was observed in the weight of the pancreas with adjacent duodenum. To assess duodenal invasion histopathologically, it was necessary to collect pancreatic tissue with the duodenum. Therefore, pancreatic weight was measured for the entire pancreas, including the adjacent duodenum (1.5 cm in length) in all mice. Consequently, pancreatic weight in this study is not suitable for evaluating pancreatic cancer. The second limitation concerns immune cells and opioid receptors. Strong opioids suppress T cells via µ‐opioid receptors expressed on T cells, and cytokines are thought to influence the expression of µ‐opioid receptors [8]. The mechanism by which high‐dose TRA administration reduces CD8^+^ T cells is unclear, but further investigation into µ‐opioid receptors expression on T cells may be necessary. The third limitation concerns CYP2D6, which is involved in the metabolism of TRA. TRA is metabolized by CYP2D6, a member of the cytochrome P450 mixed‐function oxidase system, into the active metabolite O‐desmethyl TRA and the inactive metabolite N‐desmethyl TRA [28]. The active metabolites have higher affinity for the µ‐opioid receptor. Ultimately, 30% of TRA is excreted unchanged, and 60% is excreted primarily in the urine as metabolites [28]. Humans have 27 *CYP450* functional genes, whereas mice have 72 *CYP450* functional genes; hence, it is difficult to predict the metabolic activity (otherwise known as sensitivity) of TRA [29]. The wide variety of CYP450 functional genes results in multiple genetic polymorphisms, which affect the metabolic rate of TRA [29]. In humans, of these genetic polymorphisms, an intermediate metabolizer and a poor metabolizer occur with frequencies of 10%–44% and 0.4%–6.5%, respectively [30]. These polymorphisms delay TRA metabolism, making it difficult to reduce blood TRA concentrations [31]. If these genetic polymorphisms are present, even clinically administered doses of TRA may cause stronger side effects. Therefore, one limitation of the current study is the failure to examine all polymorphisms in each mouse. Furthermore, the maximum dose of TRA for humans is 400 mg/day [22], and the dose administered to mice in this experiment was higher. However, due to differences in genetic polymorphisms and metabolic activity between humans and mice, it is difficult to convert the dose into a human equivalent. The fourth limitation is about survival time. In the present study, tumor size increased in TRA50‐treated mice, but no significant difference was observed in survival time (Supporting Figure 3B). Based on the Ki‐67 index of PDAC cells in vivo, high‐dose TRA appears to enhance the proliferative potential of these cancer cells, although TRA did not shorten survival time. This result could be related to the use of the *KPPC* mouse model, which shows an aggressive phenotype (median survival time, 57 days). Thus, use of the *KPPC* mouse model represents a limitation of the present investigation of the effects of high‐dose TRA. The fifth limitation concerns cytokine arrays and IHC analysis of the tumors. The main objective of this study was to investigate the dynamics of inflammatory immune cells within tumors induced by high‐dose TRA, and cytokine levels were measured using pooled plasma to supplement these data. The difference in sample size between the two groups was due to the exclusion of dead‐found mice and plasma samples from jaundice and hemolysis. Furthermore, in the IHC analysis of the tumors, cases with weak staining were excluded, making it impossible to analyze the tumors of all mice. These are the limitations of this study.

## 5. Conclusion

High‐dose TRA can reduce cancer‐associated pain but stimulate tumor growth by decreasing CD8^+^ T cells in vivo and increase invasive potential in vitro. Therefore, excessive amounts of TRA may contribute to tumor growth, especially in patients who have abnormalities in the metabolism of TRA [28].

## Author Contributions

Experimental design: Tomoya Kuramochi, Makoto Sano, and Takahiro Suzuki. Data acquisition/analysis: Tomoya Kuramochi, Tomoaki Itaya, and Makoto Sano. Provision of vehicle‐treated mice: Yukino Oshima. Tissue preparation: Jinsuk Kim. Discussion of data: Osamu Kitajima and Tomoaki Itaya. Writing of paper: Tomoya Kuramochi and Makoto Sano. Review of paper: Tomoaki Itaya, Hideaki Ijichi, and Takahiro Suzuki. Study supervision: Takahiro Suzuki.

## Funding

This work was funded by Japan Society for the Promotion of Science KAKENHI (JP24K12060).

## Ethics Statement

The study protocol followed the Animal Research: Reporting of In Vivo Experiments (ARRIVE) guidelines and was approved by the Nihon University School of Medicine Animal Care and Use Committee (AP21MED013 and AP22MED069).

## Consent

The authors have nothing to report.

## Conflicts of Interest

The authors declare no conflicts of interest.

## Supporting Information

Additional supporting information can be found online in the Supporting Information section.

## Supporting information


**Supporting Information 1** Supporting Figure 1. Median MGS (A) and cumulative MGS scores (B) in healthy control mice treated with TRA 50 mg/kg/day (TRA50; *n* = 4) and vehicle water (Vehicle Control; *n* = 4). Data are presented as median values. There are no significant differences between the two groups on the Mann–Whitney *U* test. Weight of the pancreas (C) and adjacent duodenum in *KPPC* mice treated with TRA at 50 mg/kg/day (TRA50) (*n* = 10) or vehicle water (*n* = 12). Data are presented as mean values. TRA50 (*n* = 10) versus vehicle (*n* = 12) (95% CI −398.4 to 605.8; *p* = 0.66) by the parametric Student’s *t*‐test. MGS, mouse grimace scale; TRA, tramadol. Supporting Figure 2. Food intake (A) and body weight (B) in *L* *S* *L* − *K* *r* *a* *s* ^*G*12*D*/+^ *;* *T* *r* *p*53^*f* *l* *o* *x*/*f* *l* *o* *x*^ *;* *P* *d* *x* − 1^*c* *r* *e*/+^ (*KPPC)* mice treated with 50 mg/kg/day TRA (TRA50; *n* = 10) or vehicle water (Vehicle; *n* = 12). Data are presented as mean values. *p*  <  0.05 by the nonparametric Mann–Whitney *U* test (∗, TRA50 vs. vehicle). TRA, tramadol. Supporting Figure 3. (A) Previous reports and current data suggest that high‐dose TRA might inhibit plasma cytokines derived from CAFs (TNF‐*α*, IL‐6, CCL3, and CCL5), M2‐like TAMs (TNF‐*α*, IL‐6, IL‐10, CCL3), CD8^+^ lymphocytes (IL‐12, IFN‐*γ*), and possible Tc9 cells (IL‐9, IL‐10). (B) Kaplan–Meier analysis shows no significant difference in survival rates of *KPPC* mice with administration of tramadol 50 mg/kg/day (TRA50; *n* = 10) and vehicle water (*n* = 12). *p* = 0.78 by the log‐rank test. TRA, tramadol. CCL, CC chemokine ligand; IL, interleukin; PDAC, pancreatic ductal adenocarcinoma; TAM, tumor‐associated macrophage; Tc9, IL‐9‐producing CD8^+^ T cells; TNF, tumor necrosis factor; TRA, tramadol. Supporting Table 1. Antibodies and conditions for immunohistochemical analysis. IHC, Immunohistochemistry. Supporting Table 2. Phenotype of *KPPC* and healthy mice with administration of 50 mg/kg/day tramadol (TRA50) and vehicle water. Supporting Table 3. Direct invasion of PDAC to other tissues/organs. Supporting Table 4. Metastasis or dissemination of PDAC.

## Data Availability

Data are available upon request from the authors.
