# Supplementary figures and images for: High‐Dose Tramadol Enhances the Proliferative and Invasive Potential of Pancreatic Ductal Adenocarcinoma in Mice Through Microenvironmental Alteration
Source: Pain Res Manag. 2026 Jun 22;2026:6594413. doi: 10.1155/prm/6594413 (PMC13284832; doi:10.1155/prm/6594413)

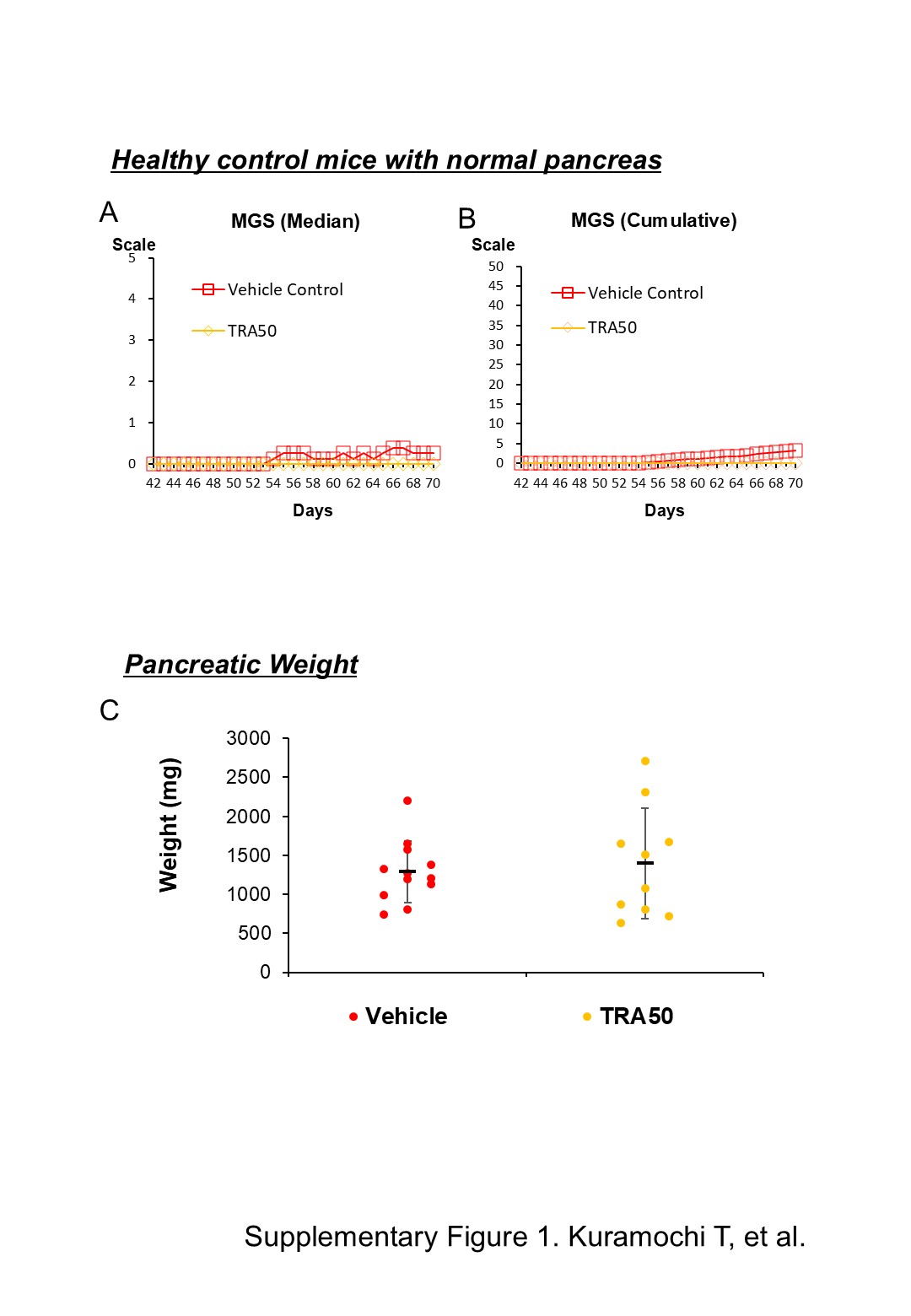

Supplement: Supplementary file 1 — Supporting Information 1 Supporting Figure 1. Median MGS (A) and cumulative MGS scores (B) in healthy control mice treated with TRA 50 mg/kg/day (TRA50; n = 4) and vehicle water (Vehicle Control; n = 4). Data are presented as median values. There are no significant differences between the two groups on the Mann–Whitney U test. Weight of the pancreas (C) and adjacent duodenum in KPPC mice treated with TRA at 50 mg/kg/day (TRA50) (n = 10) or vehicle water (n = 12). Data are presented as mean values. TRA50 (n = 10) versus vehicle (n = 12) (95% CI −398.4 to 605.8; p = 0.66) by the parametric Student’s t‐test. MGS, mouse grimace scale; TRA, tramadol. Supporting Figure 2. Food intake (A) and body weight (B) in L S L − K r a s G12D/+ ; T r p53 f l o x/f l o x ; P d x − 1 c r e/+ (KPPC) mice treated with 50 mg/kg/day TRA (TRA50; n = 10) or vehicle water (Vehicle; n = 12). Data are presented as mean values. p < 0.05 by the nonparametric Mann–Whitney U test (∗, TRA50 vs. vehicle). TRA, tramadol. Supporting Figure 3. (A) Previous reports and current data suggest that high‐dose TRA might inhibit plasma cytokines derived from CAFs (TNF‐α, IL‐6, CCL3, and CCL5), M2‐like TAMs (TNF‐α, IL‐6, IL‐10, CCL3), CD8+ lymphocytes (IL‐12, IFN‐γ), and possible Tc9 cells (IL‐9, IL‐10). (B) Kaplan–Meier analysis shows no significant difference in survival rates of KPPC mice with administration of tramadol 50 mg/kg/day (TRA50; n = 10) and vehicle water (n = 12). p = 0.78 by the log‐rank test. TRA, tramadol. CCL, CC chemokine ligand; IL, interleukin; PDAC, pancreatic ductal adenocarcinoma; TAM, tumor‐associated macrophage; Tc9, IL‐9‐producing CD8+ T cells; TNF, tumor necrosis factor; TRA, tramadol. Supporting Table 1. Antibodies and conditions for immunohistochemical analysis. IHC, Immunohistochemistry. Supporting Table 2. Phenotype of KPPC and healthy mice with administration of 50 mg/kg/day tramadol (TRA50) and vehicle water. Supporting Table 3. Direct invasion of PDAC to other tissues/organs. [file PRM-2026-6594413-s001.zip › supplementary figure (1).JPG]

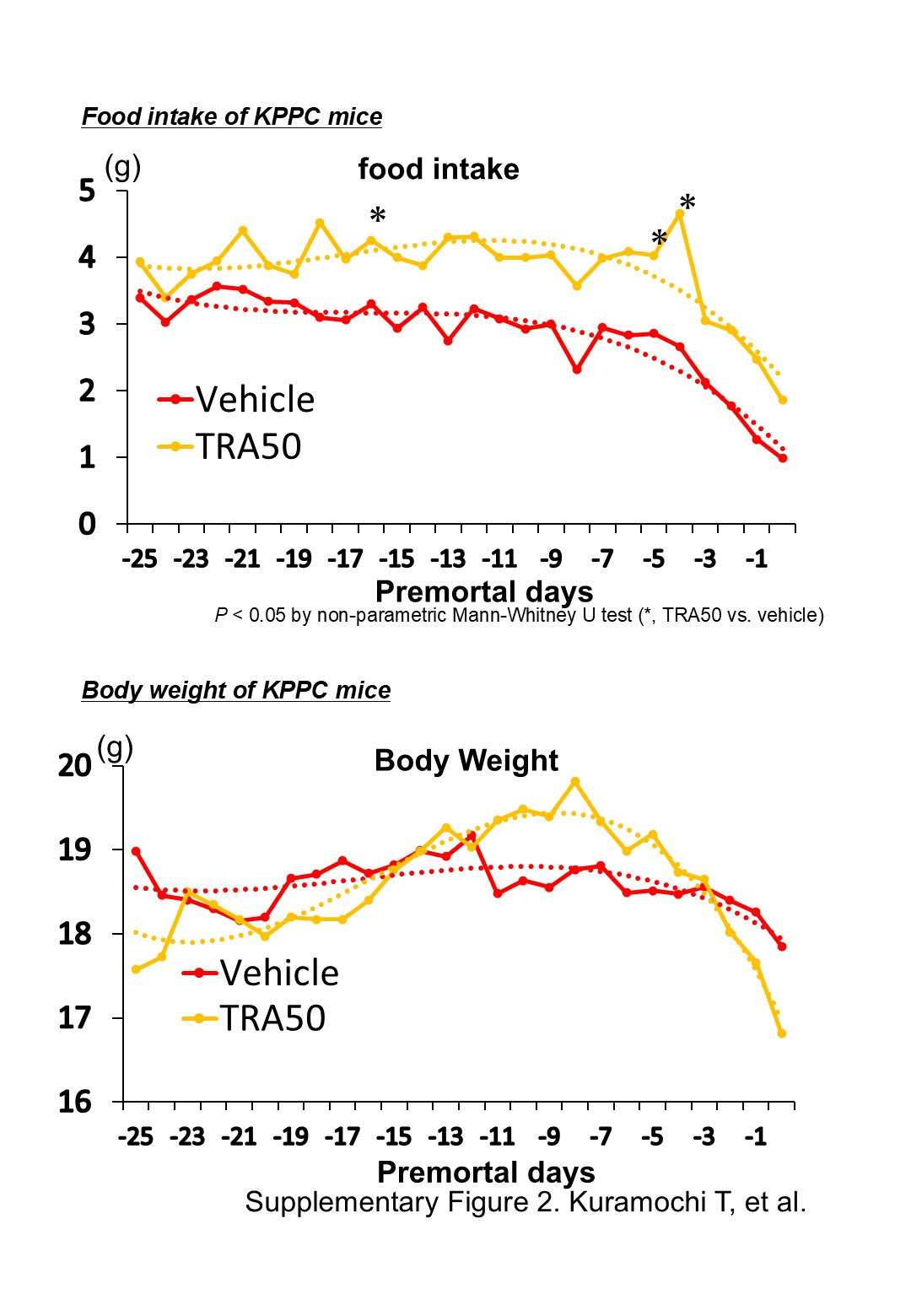

Supplement: Supplementary file 1 — Supporting Information 1 Supporting Figure 1. Median MGS (A) and cumulative MGS scores (B) in healthy control mice treated with TRA 50 mg/kg/day (TRA50; n = 4) and vehicle water (Vehicle Control; n = 4). Data are presented as median values. There are no significant differences between the two groups on the Mann–Whitney U test. Weight of the pancreas (C) and adjacent duodenum in KPPC mice treated with TRA at 50 mg/kg/day (TRA50) (n = 10) or vehicle water (n = 12). Data are presented as mean values. TRA50 (n = 10) versus vehicle (n = 12) (95% CI −398.4 to 605.8; p = 0.66) by the parametric Student’s t‐test. MGS, mouse grimace scale; TRA, tramadol. Supporting Figure 2. Food intake (A) and body weight (B) in L S L − K r a s G12D/+ ; T r p53 f l o x/f l o x ; P d x − 1 c r e/+ (KPPC) mice treated with 50 mg/kg/day TRA (TRA50; n = 10) or vehicle water (Vehicle; n = 12). Data are presented as mean values. p < 0.05 by the nonparametric Mann–Whitney U test (∗, TRA50 vs. vehicle). TRA, tramadol. Supporting Figure 3. (A) Previous reports and current data suggest that high‐dose TRA might inhibit plasma cytokines derived from CAFs (TNF‐α, IL‐6, CCL3, and CCL5), M2‐like TAMs (TNF‐α, IL‐6, IL‐10, CCL3), CD8+ lymphocytes (IL‐12, IFN‐γ), and possible Tc9 cells (IL‐9, IL‐10). (B) Kaplan–Meier analysis shows no significant difference in survival rates of KPPC mice with administration of tramadol 50 mg/kg/day (TRA50; n = 10) and vehicle water (n = 12). p = 0.78 by the log‐rank test. TRA, tramadol. CCL, CC chemokine ligand; IL, interleukin; PDAC, pancreatic ductal adenocarcinoma; TAM, tumor‐associated macrophage; Tc9, IL‐9‐producing CD8+ T cells; TNF, tumor necrosis factor; TRA, tramadol. Supporting Table 1. Antibodies and conditions for immunohistochemical analysis. IHC, Immunohistochemistry. Supporting Table 2. Phenotype of KPPC and healthy mice with administration of 50 mg/kg/day tramadol (TRA50) and vehicle water. Supporting Table 3. Direct invasion of PDAC to other tissues/organs. [file PRM-2026-6594413-s001.zip › supplementary figure (2).JPG]

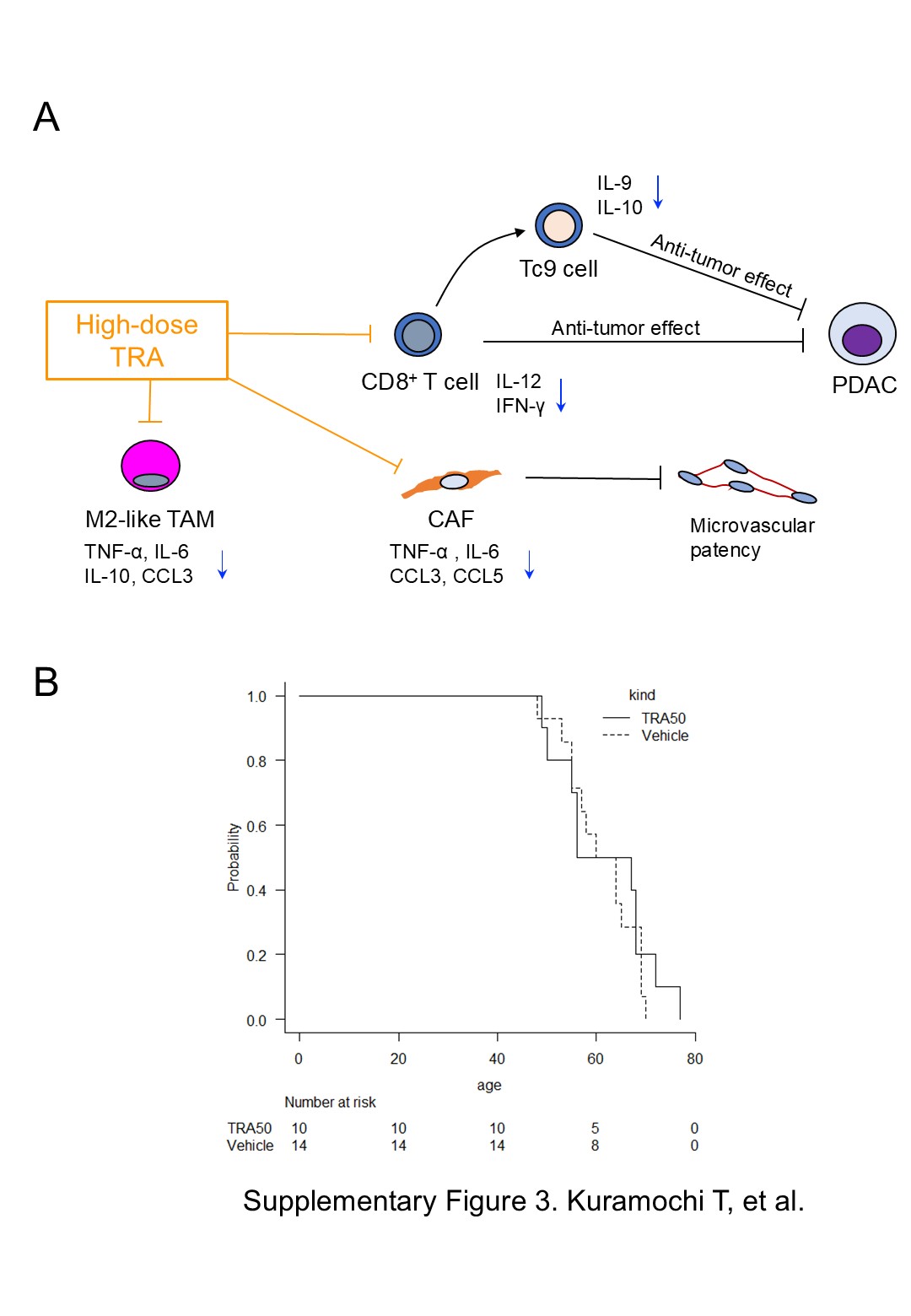

Supplement: Supplementary file 1 — Supporting Information 1 Supporting Figure 1. Median MGS (A) and cumulative MGS scores (B) in healthy control mice treated with TRA 50 mg/kg/day (TRA50; n = 4) and vehicle water (Vehicle Control; n = 4). Data are presented as median values. There are no significant differences between the two groups on the Mann–Whitney U test. Weight of the pancreas (C) and adjacent duodenum in KPPC mice treated with TRA at 50 mg/kg/day (TRA50) (n = 10) or vehicle water (n = 12). Data are presented as mean values. TRA50 (n = 10) versus vehicle (n = 12) (95% CI −398.4 to 605.8; p = 0.66) by the parametric Student’s t‐test. MGS, mouse grimace scale; TRA, tramadol. Supporting Figure 2. Food intake (A) and body weight (B) in L S L − K r a s G12D/+ ; T r p53 f l o x/f l o x ; P d x − 1 c r e/+ (KPPC) mice treated with 50 mg/kg/day TRA (TRA50; n = 10) or vehicle water (Vehicle; n = 12). Data are presented as mean values. p < 0.05 by the nonparametric Mann–Whitney U test (∗, TRA50 vs. vehicle). TRA, tramadol. Supporting Figure 3. (A) Previous reports and current data suggest that high‐dose TRA might inhibit plasma cytokines derived from CAFs (TNF‐α, IL‐6, CCL3, and CCL5), M2‐like TAMs (TNF‐α, IL‐6, IL‐10, CCL3), CD8+ lymphocytes (IL‐12, IFN‐γ), and possible Tc9 cells (IL‐9, IL‐10). (B) Kaplan–Meier analysis shows no significant difference in survival rates of KPPC mice with administration of tramadol 50 mg/kg/day (TRA50; n = 10) and vehicle water (n = 12). p = 0.78 by the log‐rank test. TRA, tramadol. CCL, CC chemokine ligand; IL, interleukin; PDAC, pancreatic ductal adenocarcinoma; TAM, tumor‐associated macrophage; Tc9, IL‐9‐producing CD8+ T cells; TNF, tumor necrosis factor; TRA, tramadol. Supporting Table 1. Antibodies and conditions for immunohistochemical analysis. IHC, Immunohistochemistry. Supporting Table 2. Phenotype of KPPC and healthy mice with administration of 50 mg/kg/day tramadol (TRA50) and vehicle water. Supporting Table 3. Direct invasion of PDAC to other tissues/organs. [file PRM-2026-6594413-s001.zip › supplementary figure (3).JPG]

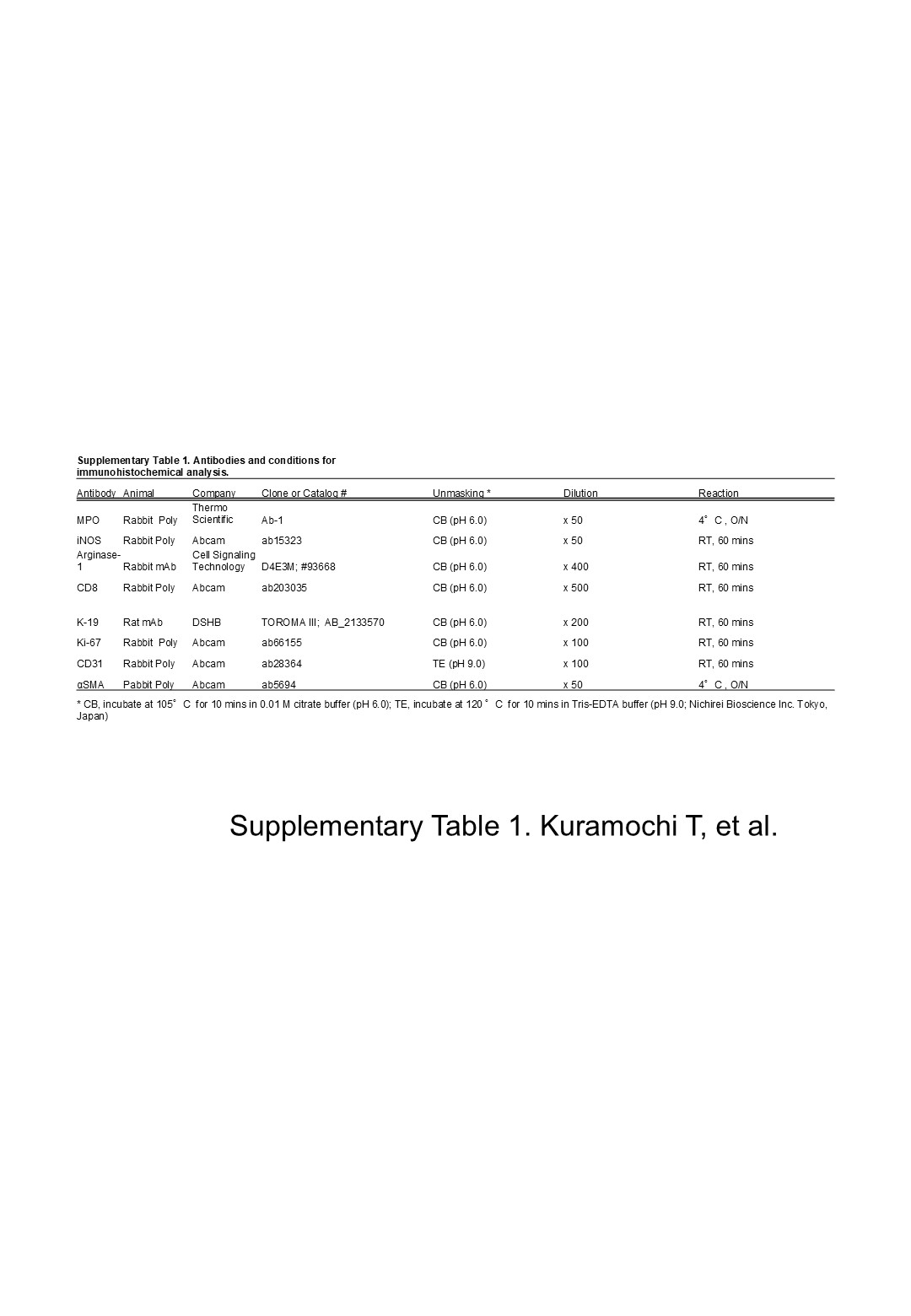

Supplement: Supplementary file 1 — Supporting Information 1 Supporting Figure 1. Median MGS (A) and cumulative MGS scores (B) in healthy control mice treated with TRA 50 mg/kg/day (TRA50; n = 4) and vehicle water (Vehicle Control; n = 4). Data are presented as median values. There are no significant differences between the two groups on the Mann–Whitney U test. Weight of the pancreas (C) and adjacent duodenum in KPPC mice treated with TRA at 50 mg/kg/day (TRA50) (n = 10) or vehicle water (n = 12). Data are presented as mean values. TRA50 (n = 10) versus vehicle (n = 12) (95% CI −398.4 to 605.8; p = 0.66) by the parametric Student’s t‐test. MGS, mouse grimace scale; TRA, tramadol. Supporting Figure 2. Food intake (A) and body weight (B) in L S L − K r a s G12D/+ ; T r p53 f l o x/f l o x ; P d x − 1 c r e/+ (KPPC) mice treated with 50 mg/kg/day TRA (TRA50; n = 10) or vehicle water (Vehicle; n = 12). Data are presented as mean values. p < 0.05 by the nonparametric Mann–Whitney U test (∗, TRA50 vs. vehicle). TRA, tramadol. Supporting Figure 3. (A) Previous reports and current data suggest that high‐dose TRA might inhibit plasma cytokines derived from CAFs (TNF‐α, IL‐6, CCL3, and CCL5), M2‐like TAMs (TNF‐α, IL‐6, IL‐10, CCL3), CD8+ lymphocytes (IL‐12, IFN‐γ), and possible Tc9 cells (IL‐9, IL‐10). (B) Kaplan–Meier analysis shows no significant difference in survival rates of KPPC mice with administration of tramadol 50 mg/kg/day (TRA50; n = 10) and vehicle water (n = 12). p = 0.78 by the log‐rank test. TRA, tramadol. CCL, CC chemokine ligand; IL, interleukin; PDAC, pancreatic ductal adenocarcinoma; TAM, tumor‐associated macrophage; Tc9, IL‐9‐producing CD8+ T cells; TNF, tumor necrosis factor; TRA, tramadol. Supporting Table 1. Antibodies and conditions for immunohistochemical analysis. IHC, Immunohistochemistry. Supporting Table 2. Phenotype of KPPC and healthy mice with administration of 50 mg/kg/day tramadol (TRA50) and vehicle water. Supporting Table 3. Direct invasion of PDAC to other tissues/organs. [file PRM-2026-6594413-s001.zip › supplementary table (1).JPG]

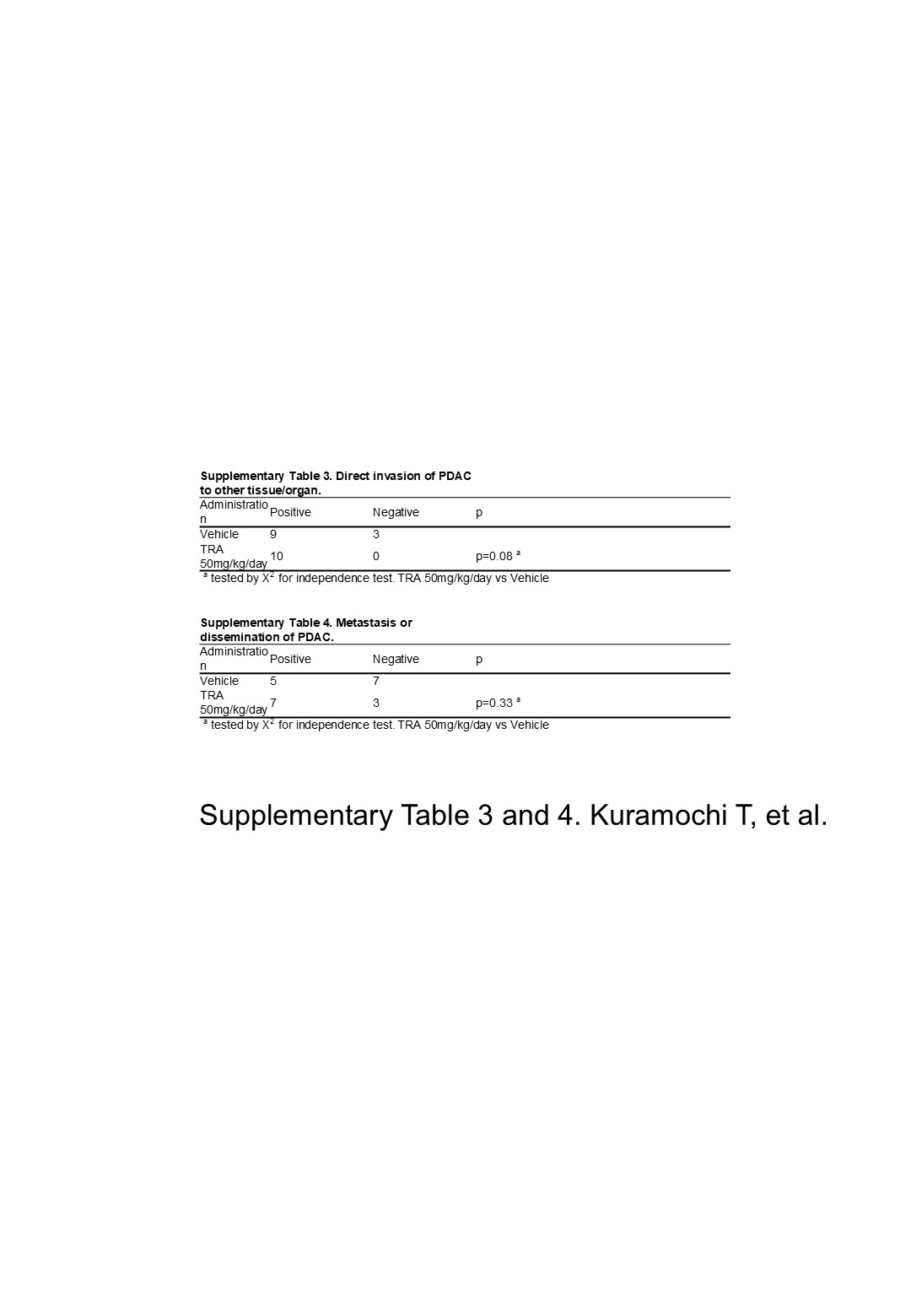

Supplement: Supplementary file 1 — Supporting Information 1 Supporting Figure 1. Median MGS (A) and cumulative MGS scores (B) in healthy control mice treated with TRA 50 mg/kg/day (TRA50; n = 4) and vehicle water (Vehicle Control; n = 4). Data are presented as median values. There are no significant differences between the two groups on the Mann–Whitney U test. Weight of the pancreas (C) and adjacent duodenum in KPPC mice treated with TRA at 50 mg/kg/day (TRA50) (n = 10) or vehicle water (n = 12). Data are presented as mean values. TRA50 (n = 10) versus vehicle (n = 12) (95% CI −398.4 to 605.8; p = 0.66) by the parametric Student’s t‐test. MGS, mouse grimace scale; TRA, tramadol. Supporting Figure 2. Food intake (A) and body weight (B) in L S L − K r a s G12D/+ ; T r p53 f l o x/f l o x ; P d x − 1 c r e/+ (KPPC) mice treated with 50 mg/kg/day TRA (TRA50; n = 10) or vehicle water (Vehicle; n = 12). Data are presented as mean values. p < 0.05 by the nonparametric Mann–Whitney U test (∗, TRA50 vs. vehicle). TRA, tramadol. Supporting Figure 3. (A) Previous reports and current data suggest that high‐dose TRA might inhibit plasma cytokines derived from CAFs (TNF‐α, IL‐6, CCL3, and CCL5), M2‐like TAMs (TNF‐α, IL‐6, IL‐10, CCL3), CD8+ lymphocytes (IL‐12, IFN‐γ), and possible Tc9 cells (IL‐9, IL‐10). (B) Kaplan–Meier analysis shows no significant difference in survival rates of KPPC mice with administration of tramadol 50 mg/kg/day (TRA50; n = 10) and vehicle water (n = 12). p = 0.78 by the log‐rank test. TRA, tramadol. CCL, CC chemokine ligand; IL, interleukin; PDAC, pancreatic ductal adenocarcinoma; TAM, tumor‐associated macrophage; Tc9, IL‐9‐producing CD8+ T cells; TNF, tumor necrosis factor; TRA, tramadol. Supporting Table 1. Antibodies and conditions for immunohistochemical analysis. IHC, Immunohistochemistry. Supporting Table 2. Phenotype of KPPC and healthy mice with administration of 50 mg/kg/day tramadol (TRA50) and vehicle water. Supporting Table 3. Direct invasion of PDAC to other tissues/organs. [file PRM-2026-6594413-s001.zip › supplementary table (3,4).JPG]
